# HPLC Method Determination of Isoliquiritin Apioside and Isoliquiritin in Rat Plasma for Application in Pharmacokinetic Study after an Oral Administration of Zhigancao Extract

**DOI:** 10.1155/2012/364013

**Published:** 2012-12-24

**Authors:** Yan-yun Yang, Liang Xu, Song-yao Hao, Yan Li, Zhen-Qiu Zhang

**Affiliations:** ^1^School of Medicine, Liaoning University of Traditional Chinese Medicine, Dalian 116600, China; ^2^Department of Medicinal Plant, Liaoning University of Traditional Chinese Medicine, Dalian 116600, China

## Abstract

A sensitive HPLC method was developed for the quantitative determination of isoliquiritin apioside (ILA) and isoliquiritin (IL) in rat plasma. After protein precipitation with acetonitrile, chloroform was used to separate lipid-soluble impurities from the plasma samples and remove acetonitrile. A chromatography was carried out on Diamonsil C18 (150 × 4.6 mm; 5 **μ**m) analytical column, using a mobile phase consisting of water (containing phosphoric acid 0.1%, v/v); acetonitrile (72 : 28, v/v) at a flow rate of 1.0 mL/min. The wavelength-switching technology was performed to determine ILA and IL at 360 nm and wogonoside (internal standard, IS) at 276 nm. The calibration curves of ILA and IL were fairly linear over the concentration ranges of 0.060–3.84 **μ**g/mL (*r* = 0.9954) and 0.075–4.80 **μ**g/mL (*r* = 0.9968), respectively. The average extract recoveries of ILA, IL, and IS were all over 80%. The precision and accuracy for all concentrations of quality controls and standards were within 15%. The lower limit of quantification (LLOQ) was 0.060 **μ**g/mL for ILA and 0.075 **μ**g/mL for IL. The method was used in pharmacokinetic study after an oral administration of Zhigancao extract to rats.

## 1. Introduction

**Figure 1:**
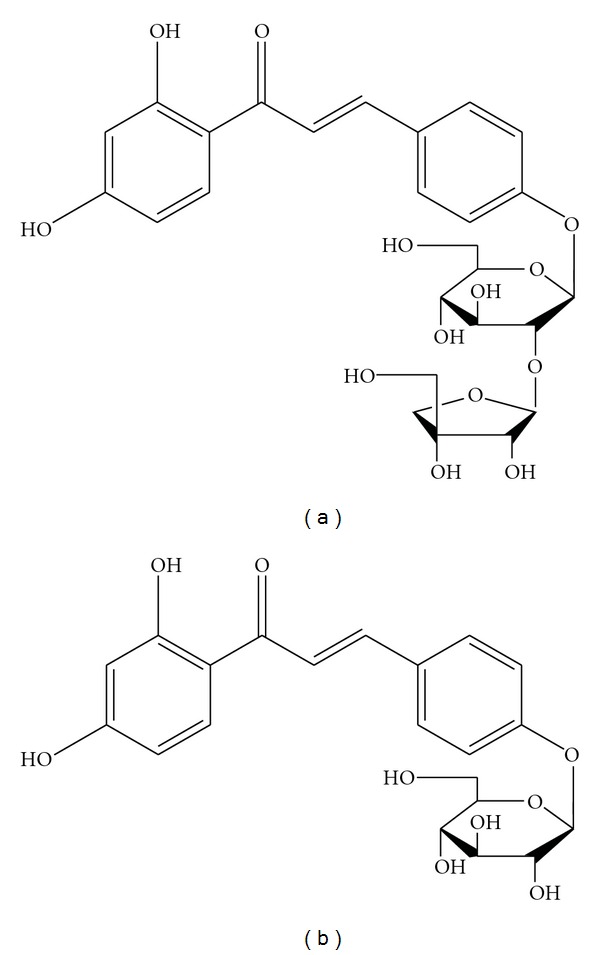
Isoliquiritin apioside (a) and isoliquiritin (b).

Zhigancao, Glycyrrhizae Radix et Rhizoma Praeparata cum Melle, originates from the processed dried roots or rhizomes of *Glycyrrhriza uralensis* Fisch., *G. inflata* Bat., or *G. glabra* L. (family Fabaceae). It is most frequently used in traditional Chinese medical formulary to harmonize all drugs and detoxify the adverse effects of herbs. Clinically, it treats disorders such as shortness of breath, fatigue, epigastria and abdominal pain, musculoskeletal and smooth muscle cramp and pain, and diarrhea [1]. Due to the numerous bioactive compounds in it such as terpenoids, saponins, polysaccharides, and flavonoids, Zhigancao has been reported to possess anti-inflammatory activities [2], antioxidative [3, 4], neuroprotective [5], antiallergic [6], anticonvulsant activities [7], and so forth. Two chalcone derivatives in Figure 1, isoliquiritin apioside (ILA) and isoliquiritin (IL), which are important ingredients in Zhigancao, are selected to research their pharmacokinetics. ILA has shown the pharmacological activity to reduce oxidative stress-induced genotoxicity [8]. And IL has been reported to have shown various pharmacological activities such as antiangiogenic effect dependent upon antitube formation [9], antidepressant-like effects in mice induced by forced swimming and tail suspension [10], and inhibitory effects on tyrosinase [11]. 

The bioavailability of drugs is the cornerstone for extending their in vitro biological functions to humans in vivo. The flavonoid glycosides show low oral bioavailability possibly because of the extensive conjugation of the free hydroxyl groups [12–14] or/and glycosides hydrolysis to the aglycones in the intestinal lumen [14–16]. The pharmacokinetic study of ILA and IL is essential for us to comprehend the bioavailability of two analytes after an oral administration of Zhigancao extrat.

To my knowledge, ILA and IL quantification method (LC-MS/MS) has been performed to study pharmacokinetics of multiple licorice flavonoids after an oral dose of Xiaochaihu-tang to rats [17], but LC-MS/MS method is simply not available in most laboratories. In this study, we established the HPLC method which was highly selective and sensitive for simultaneous quantification of ILA and IL in rat plasma. The method was used to explore the possible pharmacokinetics of ILA and IL after an oral administration of Zhigancao extract. 

## 2. Experimental

### 2.1. Reagents and Chemicals

Isoliquiritin apioside (ILA) and isoliquiritin (IL) were purchased from Xi'an Xiaocao Botanical Development Co., Ltd. (purity > 98%, Shanxi, China). Wogonoside (internal standard, IS) was obtained from National Institute for Food and Drug Control (Beijing, China). Zhigancao which was produced under the guidance of the theory of traditional Chinese medicine science was provided by Chifeng Xinzhou Traditional Chinese Medicine Co., Ltd. (Inner Mongolia, China). The material was authenticated as the dried roots of *G. uralensis* by professor Ting-guo Kang from Liaoning University of Traditional Chinese Medicine. HPLC grade acetonitrile and analytical grade chloroform were purchased from Shandong Yuwang Chemical Industry Co., Ltd. (Shandong, China). The water in the study was purified with a Milli-Q water purification system from Millipore (Bedford, USA).

### 2.2. Preparation of the Zhigancao Extract

Powdered herb materials were extracted twice under reflux condition with 70% ethanol (1 : 10, w/v) for 1 h under 100°C. The extract was filtered and evaporated. Finally, the residue was dried in vacuum drying oven at 60°C to obtain a powder state of Zhigancao extract. The contents of ILA and IL in the extract were detected by HPLC, 33.72 and 28.17 mg/g, respectively. The dried powder was stored in vacuum dryer before use.

### 2.3. Liquid Chromatographic Condition

The liquid chromatography system employed was Agilent 1100 with a variable wave length UV detector (G1314A VWD). Data was analyzed by MassHunter software (Agilent, USA). The analytical column employed was Diamonsil C18 (150 × 4.6 mm I.D., 5 **μ**m, Dikma Technologies, China) analytical column with a endcapped C18 ODS guard column. The mobile phase composed of water (containing phosphoric acid 0.1%, v/v) and acetonitrile (72 : 28, v/v) was filtered through 0.22 **μ**m Millipore membrane filter. The flow rate was 1.0 mL/min. The detection wavelength was set at 360 nm (0–9 min) and 276 nm (9–12 min). An injection volume of 20 **μ**L was optimized for final method.

### 2.4. Preparation of Standard Solution and Quality Control Samples

Stock solutions of IS, ILA and IL with concentrations of 440, 384, and 480 **μ**g/mL, respectively, were prepared in methanol, and stored at 4°C. The working solution for IS was diluted with methanol to get a final concentration of 8.80 **μ**g/mL. Stock solutions were diluted with methanol to serial standard working solutions at concentrations of 0.60, 1.20, 2.40, 4.80, 9.60, 19.2, and 38.4 **μ**g/mL for ILA, 0.75, 1.50, 3.00, 6.00, 12.0, 24.0, 36.0, and 48.0 **μ**g/mL for IL. These solutions were then added to blank plasma (1 : 10) to make standards of 0.060–3.84 **μ**g/mL for ILA and 0.075–4.80 **μ**g/mL for IL. The quality control (QC) samples which were used for the intra- and inter-day accuracy, and precision, extraction recovery and stability study, were prepared in the same way at 0.12, 0.48 and 3.07 **μ**g/mL for ILA, and 0.15, 0.60 and 3.84 **μ**g/mL for IL.

### 2.5. Sample Preparations

The 200 **μ**L of rat plasma was mixed with 20 **μ**L IS working solution. After protein was precipitated with 500 **μ**L of acetonitrile in a 1.5 mL polypropylene tube by vortexing for 3 min, the sample was centrifuged at 6,677 g for 5 min. The supernatant was transferred into a 2.0 mL tube and was added with 1,000 **μ**L of chloroform. After vortexing and centrifugation, 20 **μ**L of water phase was injected for analysis.

### 2.6. Method Validation

The analysis method was completely validated using spiked rat plasma samples, including selectivity, linearity, intra- and inter-day precision, accuracy and stability, according to the FDA guideline for method validation of bioanalytical assays [18].

The selectivity of the method was demonstrated by comparing chromatograms of blank plasma samples (without IS) obtained from rats, plasma samples spiked with the analytes and IS, and plasma samples after an oral dose. All blank plasma samples were prepared and analyzed to ensure the absence of interfering peaks.

The linearity of the method was assessed by plotting calibration curves in plasma at seven concentration levels in triplicate on three consecutive days. The lower limit of quantification (LLOQ) was defined as the lowest concentration of the calibration curve, that was measured with accuracy and precision by analyzing samples in six replicates at the concentration of 0.060 **μ**g/mL for ILA and 0.075 **μ**g/mL for IL.

Precision and accuracy were evaluated by determining QC samples at three concentrations in six replicates on the same day (intra-day accuracy and precision) and three consecutive days (inter-day accuracy and precision). Accuracy was measured by relative error (RE) and precision was evaluated by intra- and inter-day relative standard deviation (RSD).

The extraction recovery was evaluated by comparing the peak areas of the extracted QC samples at three concentrations in six replicates with those of unextracted standards that represented 100% recovery. Similarly, the recovery of IS was evaluated at a single concentration of 0.88 **μ**g/mL in the same way. 

The stability of ILA and IL in rat plasma was evaluated by comparing the mean of back-calculated concentration of the stored QC samples at three concentrations in triplicate with the spiked concentration of analytes. The QC samples treated as sample preparation were kept at room temperature for 12 h and then the stability was determined. The freeze-thaw stability was determined afterthree freeze (–20°C, 24 h) and thaw (room temperature) cycles. Long-term stability was assessed by keeping QC samples at –20°C for 15 days.

### 2.7. Applications in Pharmacokinetic Study

#### 2.7.1. Sample Collection

Female Sprague-Dawley rats (200 ± 20 g) were kept in environmental controlled breeding room for 7 days until the experiment. The rats were fasted for 12 h but allowed water ad libitum before the Zhigancao extract was orally administered at a dose of 1.21 g/kg. Orbital venous blood samples (0.5 mL) were collected before dosing, and at 0.5, 1.0, 1.5, 2, 3, 4, 6, 8, 12, 24, 36, and 48 h after an administration. After centrifuging at 6,677 g for 5 min, the plasma samples were obtained and frozen at –20°C until analysis. 

#### 2.7.2. Pharmacokinetic Analysis

HPLC analysis procedure was applied to analyze plasma concentration-time profiles of ILA and IL. Data was processed by noncompartmental method using Drug and Statistics (DAS) 2.0 software package (Chinese Pharmacological Society, Shanghai, China).

## 3. Results 

### 3.1. Method Validation

**Figure 2:**
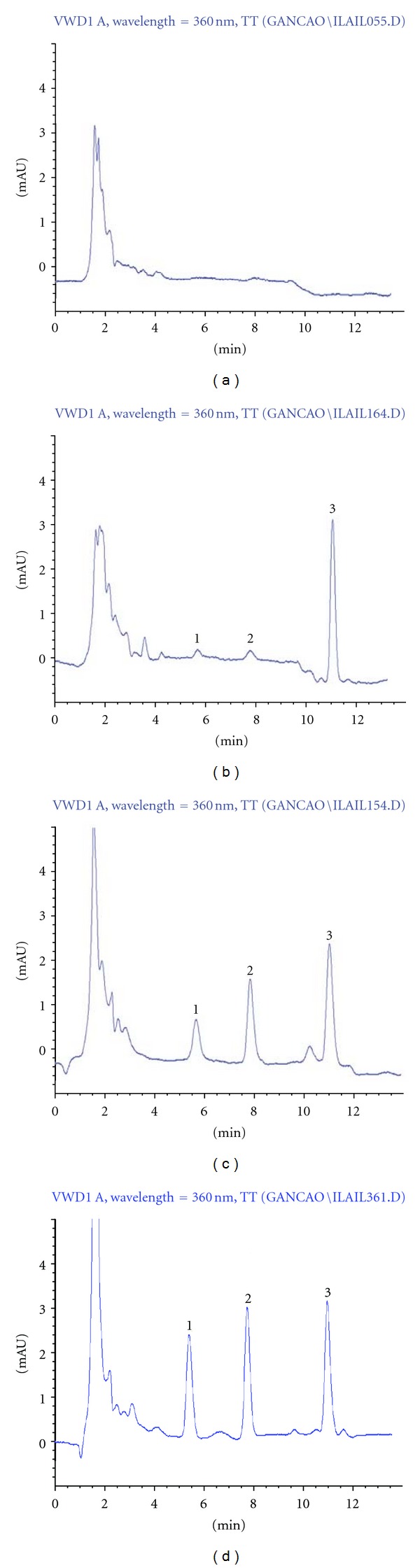
Typical HPLC chromatograms of the analytes in rat plasma (1, ILA; 2, IL; 3, IS). (a) Chromatogram of blank plasma; (b) chromatogram of plasma sample at LLOQ level (ILA 0.060 **μ**g/mL and IL 0.075 **μ**g/mL); (c) chromatogram of plasma sample at middle QC level (ILA 0.48 **μ**g/mL and IL 0.60 **μ**g/mL); (d) chromatogram of plasma sample obtained from rat (No. 2) at 1 h after an oral administration of Zhigancao extract (ILA 0.78 **μ**g/mL and IL 1.04 **μ**g/mL by calculation).

The selectivity was evaluated by analyzing blank samples, spiked samples at LLOQ, and middle QC levels, and actual samples obtained from rats after an oral administration of Zhigancao extract. The typical HPLC chromatograms were shown in Figure 2. For all blank samples, ILA, IL, and IS retention windows were free from endogenous interfering peaks. No plasma matrix effect was observed under the condition described above.

The calibration curves were linear over the concentration range of 0.060–3.84 **μ**g/mL for ILA and 0.075–4.80 **μ**g/mL for IL by weighted (1/*x* ^2^) linear least-squares regression method. The correlation coefficient values of the calibration curves were over 0.995. The REs of the back-calculated values of the standards from their nominal values were constantly within 15% for all values, including the LLOQ. The LLOQ measurement showed the respective averages 0.060 **μ**g/mL with RSD 14.5% for ILA and 0.077 **μ**g/mL with RSD 12.3% for IL. The typical chromatogram at the LLOQ was shown in Figure 2(b). Typical regression equations were calculated as follows: ILA, *y* = 1.024*x* − 0.004 (*r* = 0.9954); IL, *y* = 1.004*x* + 0.007 (*r* = 0.9968). 

The precision and accuracy data was shown in Table 1. The intra- and inter-day RSD values were lower than 10%, and the RE values were within ±5%. The results revealed satisfactory precision and accuracy of this present method.

The extraction recoveries of ILA and IL from rat plasma were 79.5 ± 4.2%, 82.5 ± 4.4%, 84.7 ± 4.0% at 1.2, 4.8, and 3.07 **μ**g/mL and 81.3 ± 4.9%, 82.3 ± 6.6%, 86.4 ± 3.4% at 1.5, 6.0, and 3.84 **μ**g/mL, respectively. The extraction recovery of IS was 81.8 ± 3.2%. These results indicated that the extraction method was suitable to extract ILA, IL, and IS from plasma.

All stability tests showed sufficient stability of ILA and IL under various test conditions. As shown in Table 2, stability of analytes showed no significant sample loss over 12 h at room temperature, three freeze-thaw cycles, and 15 days storage condition. 

### 3.2. Pharmacokinetic Study

**Figure 3:**
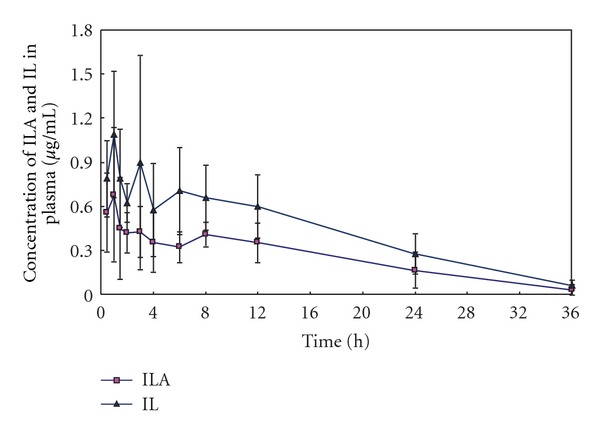
Mean plasma concentration-time profiles (*n* = 6) after an oral administration of Zhigancao extract.

The developed method was applied in pharmacokinetic study of ILA and IL in rat plasma after an oral administration of Zhigancao extract (at a dose containing 40.8 mg/kg ILA and 34.1 mg/kg IL, resp.). The mean plasma concentration-time profiles (*n* = 6) were shown in Figure 3. The pharmacokinetic parameters were shown in Table 3. The assay was sensitive enough for the determination of ILA and IL in rat plasma after an oral administration of Zhigancao extract. 

## 4. Discussion

In this study, we established an HPLC method to simultaneously quantify ILA and IL in rat plasma. Due to the stronger polarity of ILA and IL, protein precipitation was employed for the extraction of analytes from biological matrix [19]. Acetonitrile and methanol were tested as protein precipitating agent. The recovery with methanol was comparable to acetonitrile, during both processes the analyte concentration in the plasma was diluted when 2.5-fold organic solvent volume was added, so that ILA and IL couldnot be detected at a low concentration. Yet evaporating supernatant to dryness and then dissolving it in a small amount of solvent to increase the concentration would be time-consuming and would cause the loss of analyte. So removing the organic solvent from the supernatant was employed. When the supernatant was added with chloroform, a clear water phase could be obtained by precipitating the protein with acetonitrile, but no stratification could be observed when methanol was used instead of acetonitrile Therefore, plasma samples were processed by precipitating protein with acetonitrile which was then removed with chloroform. 

Wogonoside was selected as the IS because of its appropriate retention and extraction recovery. Some other compounds such as rutin, baicalin and hesperidin were also tested with the selected condition above, but without ideal results. Due to its satisfactory separation from the analytes, wogonoside was selected as the IS. Because wogonoside couldnot be detected at 360 nm at the concentration 0.88 **μ**g/mL, the wavelength was switched to 276 nm at 9 min. 

The extrapolated fraction of the AUC_0–∞_ accounted for only 5-6%, which indicated a suitability of the analytical method for ILA and IL pharmacokinetic study. These two analytes exhibited consistent tendencies in plasma concentration-time profiles and similar *T*_max⁡_, *t*_1/2*z*_ and MRT values after an oral administration of Zhigancao extract. The fact that these two compounds had the similar pharmacokinetic behavior could be tentatively attributed to their having similar structures. 

Comparing the pharmacokinetic data of ILA with that of IL, *C*_max⁡_ and AUC of ILA were lower than that of IL although there was more content of the former in Zhigancao extract. It was possible that ILA could be hydrolysis to the IL in the intestinal tract or transformed into IL after being absorbed. 

## 5. Conclusion

We have developed and validated a selective and sensitive HPLC method for simultaneous quantification of ILA and IL in rat plasma. The method was successfully applied to the pharmacokinetic study of ILA and IL in rat plasma after an oral administration of Zhigancao extract. 

## Figures and Tables

**Table 1:** Intraday and interday precision and accuracy for QC samples.

Analyte	Spiked concentration	Measured concentration	Intraday (*n* = 6)		Measured concentration	Interday (*n* = 3)	
	(*μ*g/mL)	(*μ*g/mL)	RSD (%)	RE (%)	(*μ*g/mL)	RSD (%)	RE (%)
ILA	0.12	0.12 ± 0.01	6.2	−3.6	0.12 ± 0.01	9.0	0.4
	0.48	0.46 ± 0.02	5.6	−3.4	0.48 ± 0.03	7.0	−0.8
	3.07	3.08 ± 0.16	5.4	0.2	3.02 ± 0.16	4.8	0.7

IL	0.15	0.15 ± 0.01	5.9	1.4	0.15 ± 0.01	9.7	−2.3
	0.6	0.62 ± 0.03	6.8	3.1	0.61 ± 0.04	6.8	1.0
	3.84	3.78 ± 0.24	5.0	−1.7	3.73 ± 0.18	3.5	−2.7

(Inter-day) RSD $=\left(\sqrt{\left(\sum_{i=1}^{I}{\sum_{j=1}^{n}\left(X_{ij}-{\bar{X}}_{..}\right)}^{2}-n\sum_{i=1}^{I}{\left({\bar{X}}_{i.}-{\bar{X}}_{..}\right)}^{2}\right)/\left(N-I\right)}/{\bar{X}}_{..}\right)\times 100\%$, (Inter-day) RSD $=\left(\sqrt{n\sum_{i=1}^{I}{\left({\bar{X}}_{i.}-{\bar{X}}_{..}\right)}^{2}/\left(I-1\right)}/{\bar{X}}_{..}\right)\times 100\%$.

**Table 2:** Stability of ILA and IL under various conditions in plasma (*n* = 3).

Analyte	Spiked concentration (**μ**g/mL)	Stored at room temperature for 12 h		Three freeze-thaw stability		Stored at −20°C for 15 days	
		Measured concentration (*μ*g/mL)	RE (%)	Measured concentration (*μ*g/mL)	RE (%)	Measured concentration (*μ*g/mL)	RE (%)
ILA	0.12	0.12 ± 0.001	2.7	0.12 ± 0.003	−0.5	0.12 ± 0.002	−0.5
	0.48	0.48 ± 0.008	−3.6	0.45 ± 0.018	−5.6	0.48 ± 0.002	0.07
	3.07	3.07 ± 0.028	0.03	2.91 ± 0.029	−5.2	2.97 ± 0.006	−3.2

IL	0.15	0.16 ± 0.005	4.3	0.16 ± 0.006	3.9	0.15 ± 0.004	−2.6
	0.6	0.59 ± 0.015	−1.1	0.62 ± 0.019	2.7	0.57 ± 0.010	−4.4
	3.84	3.82 ± 0.065	−0.4	3.85 ± 0.077	0.19	3.72 ± 0.157	−3.2

**Table 3:** Pharmacokinetic parameters of ILA and IL in following an oral administration of Zhigancao extract. The *C*_max⁡_ was the measured maximal concentration of ILA and IL, and the *t*_max⁡_ was the time to reach maximal concentration of ILA and IL, obtained directly from the observed value. Plasma concentrations in the terminal phase for each subject were fit to a log-linear regression by the method of least squares to obtain the elimination rate constant (*k*_*e*_). The *t*_1/2*z*_ value was calculated with the following formula: *t*_1/2_ = ln(2)/*k*_*e*_. The AUC_0–*t*_ value was the area under the concentration-time curve from 0 to 36 h (calculated using the linear trapezoidal rule), and AUC_0–*∞*_ value was calculated using the formula: AUC_0–*∞*_ = AUC_0–*t*_ + *C*_last_/*k*_*e*_, where *C*_last_ was the last measurable concentration. The mean residence time (MRT) was obtained from the formula AUMC/AUC (AUMC-area under the concentration × time curve), and MRT_0–*∞*_ was calculated using the formula: MRT_0–*∞*_ = AUMC_0–*∞*_/AUC_0–*∞*_.

Parameter	ILA	IL
*C*_max⁡_ (*μ*g/mL)	0.88 ± 0.34	1.45 ± 0.47
*T*_max⁡_ (h)	2.08 ± 2.11	1.92 ± 2.06
*t*_1/2*z*_ (h)	8.12 ± 1.72	8.56 ± 1.17
AUC_0–*t*_ (*μ*g/mL ∗ h)	8.93 ± 3.06	15.39 ± 3.91
AUC_0–*∞*_ (*μ*g/mL ∗ h)	9.49 ± 3.35	16.20 ± 4.23
MRT_0–*T*_ (h)	11.17 ± 2.72	11.53 ± 1.66
MRT_0–*∞*_ (h)	12.27 ± 4.35	13.20 ± 2.34

## References

[1] Chinese Pharmacopeia Committee (2010). *Pharmacopeia of People’s Republic of China*.

[2] Kim JK, Oh SM, Kwon HS, Oh YS, Lim SS, Shin HK (2006). Anti-inflammatory effect of roasted licorice extracts on lipopolysaccharide-induced inflammatory responses in murine macrophages. *Biochemical and Biophysical Research Communications*.

[3] Wojcikowski K, Stevenson L, Leach D, Wohlmuth H, Gobe G (2007). Antioxidant capacity of 55 medicinal herbs traditionally used to treat the urinary system: a comparison using a sequential three-solvent extraction process. *Journal of Alternative and Complementary Medicine*.

[4] Choi YJ, Lim SS, Jung JY (2008). Blockade of nitroxidative stress by roasted licorice extracts in high glucose-exposed endothelial cells. *Journal of Cardiovascular Pharmacology*.

[5] Hwang IK, Lim SS, Choi KH (2006). Neuroprotective effects of roasted licorice, not raw form, on neuronal injury in gerbil hippocampus after transient forebrain ischemia. *Acta Pharmacologica Sinica*.

[6] Majima T, Yamada T, Tega E, Sakurai H, Saiki I, Tani T (2004). Pharmaceutical evaluation of liquorice before and after roasting in mice. *Journal of Pharmacy and Pharmacology*.

[7] Yazdi A, Sardari S, Sayyah M, Ezzati MH (2011). Evaluation of the anticonvulsant activity of the leaves of *Glycyrrhiza glabra* var. glandulifera grown in Iran, as a possible renewable source for anticonvulsant compounds. *Iranian Journal of Pharmaceutical Research*.

[8] Kaur P, Kaur S, Kumar N, Singh B, Kumar S (2009). Evaluation of antigenotoxic activity of isoliquiritin apioside from *Glycyrrhiza glabra* L.. *Toxicology In Vitro*.

[9] Kobayashi S, Miyamoto T, Kimura I, Kimura M (1995). Inhibitory effect of isoliquiritin, a compound in licorice root, on angiogenesis in vivo and tube formation in vitro. *Biological and Pharmaceutical Bulletin*.

[10] Wang W, Hu X, Zhao Z (2008). Antidepressant-like effects of liquiritin and isoliquiritin from *Glycyrrhiza uralensis* in the forced swimming test and tail suspension test in mice. *Progress in Neuro-Psychopharmacology and Biological Psychiatry*.

[11] Fu B, Li H, Wang X, Lee FSC, Cui S (2005). Isolation and identification of flavonoids in licorice and a study of their inhibitory effects on tyrosinase. *Journal of Agricultural and Food Chemistry*.

[12] Manach C, Donovan JL (2004). Pharmacokinetics and metabolism of dietary flavonoids in humans. *Free Radical Research*.

[13] Williamson G, Manach C (2005). Bioavailability and bioefficacy of polyphenols in humans. II. Review of 93 intervention studies. *The American journal of clinical nutrition*.

[14] Walle T (2004). Absorption and metabolism of flavonoids. *Free Radical Biology and Medicine*.

[15] Day AJ, Cañada FJ, Díaz JC (2000). Dietary flavonoid and isoflavone glycosides are hydrolysed by the lactase site of lactase phlorizin hydrolase. *FEBS Letters*.

[16] Graefe EU, Wittig J, Mueller S (2001). Pharmacokinetics and bioavailability of quercetin glycosides in humans. *Journal of Clinical Pharmacology*.

[17] Li L, Liang S, Du F, Li C (2007). Simultaneous quantification of multiple licorice flavonoids in rat plasma. *Journal of the American Society for Mass Spectrometry*.

[18] U.S. Department of Health and Human Services (2001). *Guidance for Industry-Bioanalytical Method Validation*.

[19] Bankey S, Tapadiya G, Lamale J, Jain D, Saboo S, Khadabadi SS (2012). RP-HPLC method development and its validation for quantitative determination of rimonabant in human plasma. *Journal of Analytical Methods in Chemistry*.

